# Supplementary material for: Resolving the full spectrum of human genome variation using Linked-Reads
Source: Genome Res. 2019 Apr;29(4):635–45. doi: 10.1101/gr.234443.118 (PMC6442396; doi:10.1101/gr.234443.118)
Supplement: Supplemental Material [file supp_gr.234443.118_Supplemental_longranger-2.2.zip › longranger-2.2.tar.bz2!/lr2.2-public/lib/stan/cmdstan/stan/doc/api/html/functions__grammar__def_8hpp_source.html]

Stan: src/stan/gm/grammars/functions\_grammar\_def.hpp Source File


|  |  |
| --- | --- |
|  | Stan  2.5.0  probability, sampling & optimization |


- Main Page
- Related Pages
- Namespaces
- Classes
- Files

- File List
- File Members

All Classes Namespaces Files Functions Variables Typedefs Enumerations Enumerator Friends Macros Pages

- src
- stan
- gm
- grammars

functions\_grammar\_def.hpp

Go to the documentation of this file.

1 #ifndef STAN\_\_GM\_\_PARSER\_\_FUNCTIONS\_\_GRAMMAR\_DEF\_\_HPP\_\_

2 #define STAN\_\_GM\_\_PARSER\_\_FUNCTIONS\_\_GRAMMAR\_DEF\_\_HPP\_\_

3

4 #include <set>

5 #include <utility>

6 #include <vector>

7

8 #include <boost/spirit/include/qi.hpp>

9 #include <boost/lexical\_cast.hpp>

10 #include <boost/fusion/include/adapt\_struct.hpp>

11 #include <boost/fusion/include/std\_pair.hpp>

12 #include <boost/config/warning\_disable.hpp>

13 #include <boost/spirit/include/qi\_numeric.hpp>

14 #include <boost/spirit/include/phoenix\_core.hpp>

15 #include <boost/spirit/include/phoenix\_function.hpp>

16 #include <boost/spirit/include/phoenix\_fusion.hpp>

17 #include <boost/spirit/include/phoenix\_object.hpp>

18 #include <boost/spirit/include/phoenix\_operator.hpp>

19 #include <boost/spirit/include/phoenix\_stl.hpp>

20 #include <boost/spirit/include/support\_multi\_pass.hpp>

21 #include <boost/tuple/tuple.hpp>

22 #include <boost/variant/apply\_visitor.hpp>

23 #include <boost/variant/recursive\_variant.hpp>

24

25 #include <boost/spirit/include/version.hpp>

26 #include <boost/spirit/include/support\_line\_pos\_iterator.hpp>

27

28 #include <stan/gm/ast.hpp>

29 #include <stan/gm/grammars/functions\_grammar.hpp>

30 #include <stan/gm/grammars/statement\_grammar.hpp>

31 #include <stan/gm/grammars/whitespace\_grammar.hpp>

32

33

34 BOOST\_FUSION\_ADAPT\_STRUCT(stan::gm::function\_decl\_def,

35  (stan::gm::expr\_type, return\_type\_)

36  (std::string, name\_)

37  (std::vector<stan::gm::arg\_decl>, arg\_decls\_)

38  (stan::gm::statement, body\_) );

39

40 BOOST\_FUSION\_ADAPT\_STRUCT(stan::gm::arg\_decl,

41  (stan::gm::expr\_type, arg\_type\_)

42  (std::string, name\_)

43  (stan::gm::statement, body\_) );

44

45 namespace stan {

46

47  namespace gm {

48

49  struct validate\_non\_void\_arg\_function {

50  template <typename T1, typename T2, typename T3>

51  struct result { typedef void type; };

52  void operator()(const expr\_type& arg\_type,

53  bool& pass,

54  std::ostream& error\_msgs) const {

55  pass = !arg\_type.is\_void();

56  if (!pass)

57  error\_msgs << "Functions cannot contain void argument types; "

58  << "found void argument."

59  << std::endl;

60  }

61  };

62  boost::phoenix::function<validate\_non\_void\_arg\_function> validate\_non\_void\_arg\_f;

63

64  struct set\_void\_function {

65  template <typename T1, typename T2, typename T3, typename T4>

66  struct result { typedef void type; };

67  void operator()(const expr\_type& return\_type,

68  var\_origin& origin,

69  bool& pass,

70  std::ostream& error\_msgs) const {

71  if (return\_type.is\_void() && return\_type.num\_dims() > 0) {

72  error\_msgs << "Void return type may not have dimensions declared."

73  << std::endl;

74  pass = false;

75  return;

76  }

77  origin = return\_type.is\_void()

78  ? void\_function\_argument\_origin

79  : function\_argument\_origin;

80  }

81  };

82  boost::phoenix::function<set\_void\_function> set\_void\_function\_f;

83

84  struct set\_allows\_sampling\_origin {

85  template <typename T1, typename T2, typename T3>

86  struct result { typedef void type; };

87  void operator()(const std::string& identifier,

88  bool& allow\_sampling,

89  int& origin) const {

90  bool is\_void\_function\_origin = (origin == void\_function\_argument\_origin);

91  if (ends\_with("\_lp", identifier)) {

92  allow\_sampling = true;

93  origin = is\_void\_function\_origin

94  ? void\_function\_argument\_origin\_lp

95  : function\_argument\_origin\_lp;

96  } else if (ends\_with("\_rng", identifier)) {

97  allow\_sampling = false;

98  origin = is\_void\_function\_origin

99  ? void\_function\_argument\_origin\_rng

100  : function\_argument\_origin\_rng;

101  } else {

102  allow\_sampling = false;

103  origin = is\_void\_function\_origin

104  ? void\_function\_argument\_origin

105  : function\_argument\_origin;

106  }

107  }

108  };

109  boost::phoenix::function<set\_allows\_sampling\_origin> set\_allows\_sampling\_origin\_f;

110

111  struct validate\_declarations {

112  template <typename T1, typename T2, typename T3, typename T4>

113  struct result { typedef void type; };

114  void operator()(bool& pass,

115  std::set<std::pair<std::string,

116  function\_signature\_t> >& declared,

117  std::set<std::pair<std::string,

118  function\_signature\_t> >& defined,

119  std::ostream& error\_msgs) const {

120  using std::set;

121  using std::string;

122  using std::pair;

123  typedef set<pair<string, function\_signature\_t> >::iterator iterator\_t;

124  for (iterator\_t it = declared.begin(); it != declared.end(); ++it) {

125  if (defined.find(\*it) == defined.end()) {

126  error\_msgs <<"Function declared, but not defined."

127  << " Function name=" << (\*it).first

128  << std::endl;

129  pass = false;

130  return;

131  }

132  }

133  pass = true;

134  }

135  };

136  boost::phoenix::function<validate\_declarations> validate\_declarations\_f;

137

138  struct add\_function\_signature {

139  template <typename T1, typename T2, typename T3, typename T4, typename T5>

140  struct result { typedef void type; };

141  static bool fun\_exists(const std::set<std::pair<std::string,

142  function\_signature\_t> >& existing,

143  const std::pair<std::string,function\_signature\_t>& name\_sig) {

144  for (std::set<std::pair<std::string, function\_signature\_t> >::const\_iterator it

145  = existing.begin();

146  it != existing.end();

147  ++it)

148  if (name\_sig.first == (\*it).first

149  && name\_sig.second.second == (\*it).second.second)

150  return true; // name and arg sequences match

151  return false;

152  }

153  void operator()(const function\_decl\_def& decl,

154  bool& pass,

155  std::set<std::pair<std::string,

156  function\_signature\_t> >& functions\_declared,

157  std::set<std::pair<std::string,

158  function\_signature\_t> >& functions\_defined,

159  std::ostream& error\_msgs) const {

160

161  // build up representations

162  expr\_type result\_type(decl.return\_type\_.base\_type\_,

163  decl.return\_type\_.num\_dims\_);

164  std::vector<expr\_type> arg\_types;

165  for (size\_t i = 0; i < decl.arg\_decls\_.size(); ++i)

166  arg\_types.push\_back(expr\_type(decl.arg\_decls\_[i].arg\_type\_.base\_type\_,

167  decl.arg\_decls\_[i].arg\_type\_.num\_dims\_));

168  function\_signature\_t sig(result\_type, arg\_types);

169  std::pair<std::string, function\_signature\_t> name\_sig(decl.name\_, sig);

170

171  // check that not already declared if just declaration

172  if (decl.body\_.is\_no\_op\_statement()

173  && fun\_exists(functions\_declared,name\_sig)) {

174  error\_msgs << "Parse Error. Function already declared, name=" << decl.name\_;

175  pass = false;

176  return;

177  }

178

179  // check not already user defined

180  if (fun\_exists(functions\_defined, name\_sig)) {

181  error\_msgs << "Parse Error. Function already defined, name=" << decl.name\_;

182  pass = false;

183  return;

184  }

185

186  // check not already system defined

187  if (!fun\_exists(functions\_declared,name\_sig)

188  && function\_signatures::instance().is\_defined(decl.name\_, sig)) {

189  error\_msgs << "Parse Error. Function system defined, name=" << decl.name\_;

190  pass = false;

191  return;

192  }

193

194  // add declaration in local sets and in parser function sigs

195  if (functions\_declared.find(name\_sig) == functions\_declared.end()) {

196  functions\_declared.insert(name\_sig);

197  function\_signatures::instance()

198  .add(decl.name\_,

199  result\_type,arg\_types);

200  function\_signatures::instance()

201  .set\_user\_defined(name\_sig);

202  }

203

204  // add as definition if there's a body

205  if (!decl.body\_.is\_no\_op\_statement())

206  functions\_defined.insert(name\_sig);

207  pass = true;

208  }

209  };

210  boost::phoenix::function<add\_function\_signature> add\_function\_signature\_f;

211

212  struct validate\_return\_type {

213  template <typename T1, typename T2, typename T3>

214  struct result { typedef void type; };

215  void operator()(function\_decl\_def& decl,

216  bool& pass,

217  std::ostream& error\_msgs) const {

218  pass = decl.body\_.is\_no\_op\_statement()

219  || stan::gm::returns\_type(decl.return\_type\_, decl.body\_,

220  error\_msgs);

221  if (!pass) {

222  error\_msgs << "Improper return in body of function.";

223  return;

224  }

225

226  if (ends\_with("\_log",decl.name\_)

227  && !decl.return\_type\_.is\_primitive\_double()) {

228  pass = false;

229  error\_msgs << "Require real return type for functions ending in \_log.";

230  }

231  }

232  };

233  boost::phoenix::function<validate\_return\_type> validate\_return\_type\_f;

234

235  struct scope\_lp {

236  template <typename T1>

237  struct result { typedef void type; };

238  void operator()(variable\_map& vm) const {

239  vm.add("lp\_\_", DOUBLE\_T, local\_origin);

240  }

241  };

242  boost::phoenix::function<scope\_lp> scope\_lp\_f;

243

244

245  struct unscope\_variables {

246  template <typename T1, typename T2>

247  struct result { typedef void type; };

248  void operator()(function\_decl\_def& decl,

249  variable\_map& vm) const {

250  vm.remove("lp\_\_");

251  for (size\_t i = 0; i < decl.arg\_decls\_.size(); ++i)

252  vm.remove(decl.arg\_decls\_[i].name\_);

253  }

254  };

255  boost::phoenix::function<unscope\_variables> unscope\_variables\_f;

256

257

258  struct add\_fun\_var {

259  template <typename T1, typename T2, typename T3, typename T4>

260  struct result { typedef void type; };

261  // each type derived from base\_var\_decl gets own instance

262  void operator()(arg\_decl& decl,

263  bool& pass,

264  variable\_map& vm,

265  std::ostream& error\_msgs) const {

266  if (vm.exists(decl.name\_)) {

267  // variable already exists

268  pass = false;

269  error\_msgs << "duplicate declaration of variable, name="

270  << decl.name\_;

271

272  error\_msgs << "; attempt to redeclare as function argument";

273

274  error\_msgs << "; original declaration as ";

275  print\_var\_origin(error\_msgs,vm.get\_origin(decl.name\_));

276

277  error\_msgs << std::endl;

278  return;

279  }

280

281  pass = true;

282  vm.add(decl.name\_,

283  decl.base\_variable\_declaration(),

284  function\_argument\_origin);

285  }

286  };

287  boost::phoenix::function<add\_fun\_var> add\_fun\_var\_f;

288

289  template <typename Iterator>

290  functions\_grammar<Iterator>::functions\_grammar(variable\_map& var\_map,

291  std::stringstream& error\_msgs)

292  : functions\_grammar::base\_type(functions\_r),

293  var\_map\_(var\_map),

294  functions\_declared\_(),

295  functions\_defined\_(),

296  error\_msgs\_(error\_msgs),

297  statement\_g(var\_map\_,error\_msgs\_),

298  bare\_type\_g(var\_map\_,error\_msgs\_)

299  {

300  using boost::spirit::qi::\_1;

301  using boost::spirit::qi::char\_;

302  using boost::spirit::qi::eps;

303  using boost::spirit::qi::lexeme;

304  using boost::spirit::qi::lit;

305  using boost::spirit::qi::no\_skip;

306  using boost::spirit::qi::\_pass;

307  using boost::spirit::qi::\_val;

308

309  using boost::spirit::qi::labels::\_a;

310  using boost::spirit::qi::labels::\_b;

311  using boost::spirit::qi::labels::\_r1;

312  using boost::spirit::qi::labels::\_r2;

313

314  using boost::spirit::qi::on\_error;

315  using boost::spirit::qi::fail;

316  using boost::spirit::qi::rethrow;

317  using namespace boost::spirit::qi::labels;

318

319  functions\_r.name("function declarations and definitions");

320  functions\_r

321  %= lit("functions")

322  >> lit('{')

323  >> \*function\_r

324  >> lit('}')[ validate\_declarations\_f(\_pass,

325  boost::phoenix::ref(functions\_declared\_),

326  boost::phoenix::ref(functions\_defined\_),

327  boost::phoenix::ref(error\_msgs\_) ) ]

328  ;

329  // locals: \_a = allow sampling, \_b = origin (function, rng/lp)

330  function\_r.name("function declaration or definition");

331  function\_r

332  %= bare\_type\_g[ set\_void\_function\_f(\_1,\_b, \_pass,

333  boost::phoenix::ref(error\_msgs\_)) ]

334  > identifier\_r[ set\_allows\_sampling\_origin\_f(\_1,\_a,\_b) ]

335  > lit('(')

336  > arg\_decls\_r

337  > lit(')')

338  > eps [ scope\_lp\_f(boost::phoenix::ref(var\_map\_)) ]

339  > statement\_g(\_a,\_b,true)

340  > eps [ unscope\_variables\_f(\_val,

341  boost::phoenix::ref(var\_map\_)) ]

342  > eps [ validate\_return\_type\_f(\_val,\_pass,

343  boost::phoenix::ref(error\_msgs\_)) ]

344  > eps [ add\_function\_signature\_f(\_val,\_pass,

345  boost::phoenix::ref(functions\_declared\_),

346  boost::phoenix::ref(functions\_defined\_),

347  boost::phoenix::ref(error\_msgs\_) ) ]

348  ;

349

350  arg\_decls\_r.name("function argument declaration sequence");

351  arg\_decls\_r

352  %= arg\_decl\_r % ','

353  | eps

354  ;

355

356  arg\_decl\_r.name("function argument declaration");

357  arg\_decl\_r

358  %= bare\_type\_g [ validate\_non\_void\_arg\_f(\_1, \_pass,

359  boost::phoenix::ref(error\_msgs\_)) ]

360  > identifier\_r

361  > eps[ add\_fun\_var\_f(\_val,\_pass,

362  boost::phoenix::ref(var\_map\_),

363  boost::phoenix::ref(error\_msgs\_)) ]

364  ;

365

366  identifier\_r.name("identifier");

367  identifier\_r

368  %= lexeme[char\_("a-zA-Z")

369  >> \*char\_("a-zA-Z0-9\_.")];

370

371  }

372

373  }

374 }

375 #endif

376

stan::gm::functions\_grammar::bare\_type\_g

bare\_type\_grammar< Iterator > bare\_type\_g

**Definition:** functions\_grammar.hpp:40

stan::gm::validate\_non\_void\_arg\_function

**Definition:** functions\_grammar\_def.hpp:49

stan::gm::validate\_declarations::result::type

void type

**Definition:** functions\_grammar\_def.hpp:113

stan::gm::statement

**Definition:** ast.hpp:634

stan::gm::statement::is\_no\_op\_statement

bool is\_no\_op\_statement() const

**Definition:** ast\_def.cpp:1286

stan::gm::ends\_with

bool ends\_with(const std::string &suffix, const std::string &s)

**Definition:** ast\_def.cpp:1477

stan::gm::scope\_lp::result

**Definition:** functions\_grammar\_def.hpp:237

stan::gm::functions\_grammar::arg\_decls\_r

boost::spirit::qi::rule< Iterator, std::vector< arg\_decl >), whitespace\_grammar< Iterator > > arg\_decls\_r

**Definition:** functions\_grammar.hpp:61

stan::gm::function\_signatures::add

void add(const std::string &name, const expr\_type &result\_type, const std::vector< expr\_type > &arg\_types)

**Definition:** ast\_def.cpp:171

stan::gm::expr\_type::base\_type\_

base\_expr\_type base\_type\_

**Definition:** ast.hpp:75

stan::gm::variable\_map::add

void add(const std::string &name, const base\_var\_decl &base\_decl, const var\_origin &vo)

**Definition:** ast\_def.cpp:1007

stan::gm::function\_signatures::is\_defined

bool is\_defined(const std::string &name, const function\_signature\_t &sig)

**Definition:** ast\_def.cpp:163

stan::gm::validate\_non\_void\_arg\_function::result::type

void type

**Definition:** functions\_grammar\_def.hpp:51

stan::gm::add\_function\_signature::result

**Definition:** functions\_grammar\_def.hpp:140

stan::gm::DOUBLE\_T

const int DOUBLE\_T

**Definition:** ast.hpp:66

stan::gm::arg\_decl::name\_

std::string name\_

**Definition:** ast.hpp:769

stan::gm::function\_signatures::instance

static function\_signatures & instance()

**Definition:** ast\_def.cpp:143

stan::gm::local\_origin

const int local\_origin

**Definition:** ast.hpp:418

stan::gm::unscope\_variables::result

**Definition:** functions\_grammar\_def.hpp:247

stan::gm::add\_function\_signature

**Definition:** functions\_grammar\_def.hpp:138

stan::gm::validate\_non\_void\_arg\_function::result

**Definition:** functions\_grammar\_def.hpp:51

functions\_grammar.hpp

stan::gm::function\_signatures::set\_user\_defined

void set\_user\_defined(const std::pair< std::string, function\_signature\_t > &name\_sig)

**Definition:** ast\_def.cpp:150

stan::gm::validate\_declarations\_f

boost::phoenix::function< validate\_declarations > validate\_declarations\_f

**Definition:** functions\_grammar\_def.hpp:136

stan::gm::expr\_type::is\_primitive\_double

bool is\_primitive\_double() const

**Definition:** ast\_def.cpp:101

stan::gm::unscope\_variables::operator()

void operator()(function\_decl\_def &decl, variable\_map &vm) const

**Definition:** functions\_grammar\_def.hpp:248

stan::gm::print\_var\_origin

void print\_var\_origin(std::ostream &o, const var\_origin &vo)

**Definition:** ast\_def.cpp:944

stan::gm::validate\_return\_type

**Definition:** functions\_grammar\_def.hpp:212

stan::gm::function\_decl\_def

**Definition:** ast.hpp:776

stan::gm::function\_argument\_origin

const int function\_argument\_origin

**Definition:** ast.hpp:419

statement\_grammar.hpp

stan::gm::add\_function\_signature::fun\_exists

static bool fun\_exists(const std::set< std::pair< std::string, function\_signature\_t > > &existing, const std::pair< std::string, function\_signature\_t > &name\_sig)

**Definition:** functions\_grammar\_def.hpp:141

stan::gm::validate\_declarations::operator()

void operator()(bool &pass, std::set< std::pair< std::string, function\_signature\_t > > &declared, std::set< std::pair< std::string, function\_signature\_t > > &defined, std::ostream &error\_msgs) const

**Definition:** functions\_grammar\_def.hpp:114

stan::return\_type

Metaprogram to calculate the base scalar return type resulting from promoting all the scalar types of...

**Definition:** traits.hpp:398

stan::gm::void\_function\_argument\_origin\_rng

const int void\_function\_argument\_origin\_rng

**Definition:** ast.hpp:424

stan::gm::function\_argument\_origin\_rng

const int function\_argument\_origin\_rng

**Definition:** ast.hpp:421

stan::gm::scope\_lp::result::type

void type

**Definition:** functions\_grammar\_def.hpp:237

stan::gm::set\_void\_function\_f

boost::phoenix::function< set\_void\_function > set\_void\_function\_f

**Definition:** functions\_grammar\_def.hpp:82

stan::gm::add\_fun\_var\_f

boost::phoenix::function< add\_fun\_var > add\_fun\_var\_f

**Definition:** functions\_grammar\_def.hpp:287

stan::gm::set\_allows\_sampling\_origin::result

**Definition:** functions\_grammar\_def.hpp:86

stan::gm::set\_void\_function::result::type

void type

**Definition:** functions\_grammar\_def.hpp:66

stan::gm::unscope\_variables\_f

boost::phoenix::function< unscope\_variables > unscope\_variables\_f

**Definition:** functions\_grammar\_def.hpp:255

stan::gm::expr\_type::num\_dims\_

size\_t num\_dims\_

**Definition:** ast.hpp:76

stan::gm::unscope\_variables

**Definition:** functions\_grammar\_def.hpp:245

stan::gm::validate\_return\_type::result

**Definition:** functions\_grammar\_def.hpp:214

stan::gm::functions\_grammar::arg\_decl\_r

boost::spirit::qi::rule< Iterator, arg\_decl(), whitespace\_grammar< Iterator > > arg\_decl\_r

**Definition:** functions\_grammar.hpp:66

stan::gm::variable\_map

**Definition:** ast.hpp:439

stan::gm::scope\_lp::operator()

void operator()(variable\_map &vm) const

**Definition:** functions\_grammar\_def.hpp:238

stan::gm::set\_allows\_sampling\_origin\_f

boost::phoenix::function< set\_allows\_sampling\_origin > set\_allows\_sampling\_origin\_f

**Definition:** functions\_grammar\_def.hpp:109

stan::gm::expr\_type

**Definition:** ast.hpp:74

stan::gm::functions\_grammar::var\_map\_

variable\_map & var\_map\_

**Definition:** functions\_grammar.hpp:27

stan::gm::add\_function\_signature::operator()

void operator()(const function\_decl\_def &decl, bool &pass, std::set< std::pair< std::string, function\_signature\_t > > &functions\_declared, std::set< std::pair< std::string, function\_signature\_t > > &functions\_defined, std::ostream &error\_msgs) const

**Definition:** functions\_grammar\_def.hpp:153

stan::gm::void\_function\_argument\_origin\_lp

const int void\_function\_argument\_origin\_lp

**Definition:** ast.hpp:423

stan::gm::validate\_return\_type\_f

boost::phoenix::function< validate\_return\_type > validate\_return\_type\_f

**Definition:** functions\_grammar\_def.hpp:233

stan::gm::expr\_type::num\_dims

size\_t num\_dims() const

**Definition:** ast\_def.cpp:114

stan::gm::functions\_grammar::function\_r

boost::spirit::qi::rule< Iterator, boost::spirit::qi::locals< bool, int >, function\_decl\_def(), whitespace\_grammar< Iterator > > function\_r

**Definition:** functions\_grammar.hpp:56

stan::gm::scope\_lp

**Definition:** functions\_grammar\_def.hpp:235

BOOST\_FUSION\_ADAPT\_STRUCT

BOOST\_FUSION\_ADAPT\_STRUCT(stan::gm::function\_decl\_def,(stan::gm::expr\_type, return\_type\_)(std::string, name\_)(std::vector< stan::gm::arg\_decl >, arg\_decls\_)(stan::gm::statement, body\_))

stan::gm::set\_void\_function::operator()

void operator()(const expr\_type &return\_type, var\_origin &origin, bool &pass, std::ostream &error\_msgs) const

**Definition:** functions\_grammar\_def.hpp:67

stan::gm::function\_decl\_def::arg\_decls\_

std::vector< arg\_decl > arg\_decls\_

**Definition:** ast.hpp:784

stan::gm::validate\_declarations

**Definition:** functions\_grammar\_def.hpp:111

stan::gm::functions\_grammar::functions\_grammar

functions\_grammar(variable\_map &var\_map, std::stringstream &error\_msgs)

**Definition:** functions\_grammar\_def.hpp:290

stan::gm::functions\_grammar::statement\_g

statement\_grammar< Iterator > statement\_g

**Definition:** functions\_grammar.hpp:39

stan::gm::void\_function\_argument\_origin

const int void\_function\_argument\_origin

**Definition:** ast.hpp:422

stan::gm::add\_fun\_var

**Definition:** functions\_grammar\_def.hpp:258

stan::gm::function\_decl\_def::name\_

std::string name\_

**Definition:** ast.hpp:783

stan::gm::functions\_grammar::functions\_declared\_

std::set< std::pair< std::string, function\_signature\_t > > functions\_declared\_

**Definition:** functions\_grammar.hpp:32

stan::gm::add\_function\_signature::result::type

void type

**Definition:** functions\_grammar\_def.hpp:140

stan::gm::functions\_grammar::functions\_r

boost::spirit::qi::rule< Iterator, std::vector< function\_decl\_def >), whitespace\_grammar< Iterator > > functions\_r

**Definition:** functions\_grammar.hpp:50

stan::gm::add\_fun\_var::operator()

void operator()(arg\_decl &decl, bool &pass, variable\_map &vm, std::ostream &error\_msgs) const

**Definition:** functions\_grammar\_def.hpp:262

stan::gm::add\_fun\_var::result

**Definition:** functions\_grammar\_def.hpp:260

stan::gm::functions\_grammar::identifier\_r

boost::spirit::qi::rule< Iterator, std::string(), whitespace\_grammar< Iterator > > identifier\_r

**Definition:** functions\_grammar.hpp:71

stan::gm::set\_allows\_sampling\_origin::result::type

void type

**Definition:** functions\_grammar\_def.hpp:86

ast.hpp

stan::gm::set\_allows\_sampling\_origin

**Definition:** functions\_grammar\_def.hpp:84

stan::gm::add\_function\_signature\_f

boost::phoenix::function< add\_function\_signature > add\_function\_signature\_f

**Definition:** functions\_grammar\_def.hpp:210

stan::gm::functions\_grammar::functions\_defined\_

std::set< std::pair< std::string, function\_signature\_t > > functions\_defined\_

**Definition:** functions\_grammar.hpp:34

stan::gm::function\_decl\_def::return\_type\_

expr\_type return\_type\_

**Definition:** ast.hpp:782

stan::gm::variable\_map::remove

void remove(const std::string &name)

**Definition:** ast\_def.cpp:1012

stan::gm::validate\_declarations::result

**Definition:** functions\_grammar\_def.hpp:113

stan::gm::arg\_decl::base\_variable\_declaration

base\_var\_decl base\_variable\_declaration()

**Definition:** ast\_def.cpp:403

whitespace\_grammar.hpp

stan::gm::function\_argument\_origin\_lp

const int function\_argument\_origin\_lp

**Definition:** ast.hpp:420

stan::gm::scope\_lp\_f

boost::phoenix::function< scope\_lp > scope\_lp\_f

**Definition:** functions\_grammar\_def.hpp:242

stan::gm::set\_void\_function::result

**Definition:** functions\_grammar\_def.hpp:66

stan::gm::functions\_grammar::error\_msgs\_

std::stringstream & error\_msgs\_

**Definition:** functions\_grammar.hpp:36

stan::gm::validate\_non\_void\_arg\_function::operator()

void operator()(const expr\_type &arg\_type, bool &pass, std::ostream &error\_msgs) const

**Definition:** functions\_grammar\_def.hpp:52

stan::gm::add\_fun\_var::result::type

void type

**Definition:** functions\_grammar\_def.hpp:260

stan::gm::validate\_return\_type::result::type

void type

**Definition:** functions\_grammar\_def.hpp:214

stan::gm::function\_decl\_def::body\_

statement body\_

**Definition:** ast.hpp:785

stan::gm::set\_void\_function

**Definition:** functions\_grammar\_def.hpp:64

stan::gm::functions\_grammar

**Definition:** functions\_grammar.hpp:21

stan::gm::variable\_map::exists

bool exists(const std::string &name) const

**Definition:** ast\_def.cpp:988

stan::gm::set\_allows\_sampling\_origin::operator()

void operator()(const std::string &identifier, bool &allow\_sampling, int &origin) const

**Definition:** functions\_grammar\_def.hpp:87

stan::gm::validate\_return\_type::operator()

void operator()(function\_decl\_def &decl, bool &pass, std::ostream &error\_msgs) const

**Definition:** functions\_grammar\_def.hpp:215

stan::gm::unscope\_variables::result::type

void type

**Definition:** functions\_grammar\_def.hpp:247

stan::gm::validate\_non\_void\_arg\_f

boost::phoenix::function< validate\_non\_void\_arg\_function > validate\_non\_void\_arg\_f

**Definition:** functions\_grammar\_def.hpp:62

stan::gm::returns\_type

bool returns\_type(const expr\_type &return\_type, const statement &statement, std::ostream &error\_msgs)

**Definition:** ast\_def.cpp:515

stan::gm::var\_origin

int var\_origin

**Definition:** ast.hpp:411

stan::gm::variable\_map::get\_origin

var\_origin get\_origin(const std::string &name) const

**Definition:** ast\_def.cpp:1002

stan::gm::expr\_type::is\_void

bool is\_void() const

**Definition:** ast\_def.cpp:108

stan::gm::function\_signature\_t

std::pair< expr\_type, std::vector< expr\_type > > function\_signature\_t

**Definition:** ast.hpp:104

stan::gm::arg\_decl

**Definition:** ast.hpp:767


---

[ Stan Home Page ]

*© 2011–2014,
Stan Development Team.*
